# Efficient Convolutional Network Model Incorporating a Multi-Attention Mechanism for Individual Recognition of Holstein Dairy Cows

**DOI:** 10.3390/ani15081173

**Published:** 2025-04-19

**Authors:** Xiaoli Ma, Youxin Yu, Wenbo Zhu, Yu Liu, Linhui Gan, Xiaoping An, Honghui Li, Buyu Wang

**Affiliations:** 1 College of Computer and Information Engineering, Inner Mongolia Agricultural University, Hohhot 010018, China; maxiaoli@emails.imau.edu.cn (X.M.); yxyu1@emails.imau.edu.cn (Y.Y.); zhuwbcs@emails.imau.edu.cn (W.Z.); liuyu598850462@emails.imau.edu.cn (Y.L.); ganlh@emails.imau.edu.cn (L.G.); lihh@imau.edu.cn (H.L.); 2 Key Laboratory of Smart Animal Husbandry, Universities of Inner Mongolia Autonomous Region, Hohhot 010018, China; anxiaoping@imau.edu.cn; 3 College of Animal Science, Inner Mongolia Agricultural University, Hohhot 010018, China

**Keywords:** Holstein cow, individual recognition, LightCBAM, CowBackNet, Grad-CAM

## Abstract

Individual recognition of Holstein cows is the basis for realizing precision dairy farming. Current machine vision individual recognition systems usually rely on fixed vertical illumination and top-view camera perspectives or require complex camera systems, and these requirements limit their promotion in practical applications. To solve this problem, a lightweight Holstein cow individual recognition feature extraction network named CowBackNet is designed in this paper. This network is not affected by camera angle and lighting changes and is suitable for farm environments. Secondly, a fusion multi-attention mechanism approach was adopted to integrate the attention mechanism, inverse residual structure, and depth-separable convolution technique to design a new feature extraction module, LightCBAM. This module was placed in the corresponding layer of CowBackNet to enhance the model’s ability to extract the key features of the cow’s back image from different viewpoints. In addition, the CowBack dataset was constructed in this study to verify the model’s ability to be applied in real scenarios, containing Holstein cowback images in real production environments under different viewpoints. The experimental results show that when using CowBackNet as a feature extraction network, the recognition accuracy reaches 88.30%, FLOPs are 0.727 G, and the model size is only 6.096 MB. Compared with the classical EfficientNetV2, the accuracy of CowBackNet is improved by 11.69%, the FLOPs are reduced by 0.001 G, and the number of parameters is also reduced by 14.6%. Therefore, the model developed in this paper shows good robustness in shooting angle, light change, and real production data, which not only improves the recognition accuracy but also optimizes the computational efficiency of the model, which is of great practical application value for realizing precision farming.

## Simple Summary

The accurate identification of Holstein cows is essential for precision dairy farming, facilitating improved livestock management. To achieve this, using images from different viewpoints is vital, as it captures unique features that vary with camera angles and lighting conditions. This paper introduces a lightweight feature extraction network that enhances recognition accuracy by processing cowback images from various perspectives. A dataset of real production environment images was created for validation. Experimental results show that the network achieves 88.30% recognition accuracy while reducing computational demands compared to existing models, improving accuracy by 11.69%. This approach effectively addresses recognition challenges, enhancing precision dairy farming’s practicality and efficiency.

## 1. Introduction

With the acceleration of the modernization process of dairy farming, the demand for accurate production in farms is becoming increasingly prominent [1]. Holstein cows, as the core population of dairy farming, have individual identity markers that are inefficient and lack sufficient accuracy if relying on manual methods. The accuracy of these data directly affects the productivity and economic benefits of dairy farms. Therefore, developing a fast and accurate intelligent identification system and applying it practically in the cattle farm environment is particularly important. Individual identification of Holstein dairy cows is a core aspect of realizing precision farming. It plays a crucial role in automated management, such as body condition monitoring [2], disease early warning [3], and estrus detection [4].

Among traditional identification methods, manual identification is inefficient and prone to errors, alongside radio frequency identification (RFID) [5]. Whereas RFID has been widely used in Holstein dairy farming [6], a common method of identification is through the installation of electronic ear tags. Still, ear tags are easily lost during Holstein cow activities [7], which increases the cost of replacing ear tags and may lead to stress in cows [8]. Therefore, RFID individual identification methods for Holstein cows may trigger animal stress and pose high economic costs and potential animal welfare problems.

Holstein cows and their crossbred progeny have black and white patchy coats, also known as black and white flower cows. Different individuals display unique textural features that can be considered identity traits, and each Holstein cow is uniquely identified by image recognition techniques [9]. In livestock farming, computer vision technology is increasingly becoming pivotal for identification, owing to its notable benefits: non-intrusiveness, absence of stress induction, and economic efficiency. This technology implements the automatic identification of individual Holstein cows utilizing a vision acquisition device and a computing device without needing additional equipment. It can fully use the textural features extracted from the facial features, the uniqueness of the muzzle pattern of Holstein cows [10,11], and the details of the body contour. This technique not only safeguards the welfare of animals but also improves the efficiency and accuracy of individual identification [12,13].

The research on individual identification of Holstein dairy cows based on computer vision technology has made significant progress, providing strong technical support for Holstein dairy cow management, health monitoring, and improving production efficiency. The application of this technology not only enhances the identification efficiency but also reduces the error rate of manual operation, injecting new vitality into the development of modern animal husbandry. For example, Fu et al. [14] introduced a non-invasive system for individual Holstein cow recognition, utilizing an enhanced ResNet50 architecture integrated with the Ghost module and convolutional block attention mechanism. The method significantly reduces the number of parameters and the model size while maintaining high accuracy. Yang et al. [15] proposed an individual cow recognition method fusing RetinaFace and an improved version of FaceNet, which achieves, through MobileNet-enhanced RetinaFace and joint optimization of cross-entropy loss and ternary loss, an accuracy of 99.50% accuracy and 83.60% on the test set. Unlike the previous recognition approach using single features, Hu et al. [16] attained a recognition accuracy of 98.36% on a dataset comprising 93 cow side views by isolating features from the head, torso, and legs, treating them as distinct components, and subsequently amalgamating depth-based features from each component via an advanced depth part feature fusion technique.

The aforementioned research indicates that individual Holstein cows can be identified using their heads and trunks; however, the practical applications of these methods are somewhat constrained [17,18]. Images of the head and side of Holstein cows taken at different angles are prone to distortion and occlusion and, thus, unsuitable for individual recognition on large-scale farms. A feasible method is to acquire Holstein cowback images through a top-view angle; for this reason, Xiao et al. [19] proposed a method to recognize individual Holstein cows using an improved Mask R-CNN model and SVM classifier. The technique improved the accuracy of Holstein cow recognition to 98.67% by top-view image analysis, effectively solved the challenge of individual Holstein cow recognition in a free environment, and demonstrated the potential of application in accurate Holstein cow management. Similarly, Xu et al. [20] proposed a BottleNet Transformer (BoTNet) model combining graph sampling and counterfactual attentional learning, which optimizes feature capture in the back pattern region of Holstein cows, outperforms existing techniques on public datasets, has better generalization ability, and effectively solves the problem of individual recognition in large-scale farms. Wang et al. [21] proposed an open-set recognition method integrating spatial feature transformation and metric learning for the issue of directional diversity and partial occlusion in the recognition of individual Holstein cows from a top-view angle, which achieved 94.58% recognition accuracy under open set and improved the performance of recognition of partially visible Holstein cows by optimizing the deep feature extraction module and the attention mechanism. These studies show that computer vision technology based on target detection and image recognition has demonstrated significant advantages in Holstein cow recognition applications, which not only avoid stressing Holstein cows but also significantly reduce labor costs and have good prospects for promotion and application [22].

However, due to the fact that the majority of Holstein cowback images are captured from an overhead perspective in practical farming environments, factors such as cow movement and variations in lighting conditions can result in inadequate extraction of image features, thereby diminishing the precision of the recognition process. Aiming at the problems in the traditional top-view recognition method, this study proposes a recognition scheme that uses different viewpoints to acquire Holstein cowback images and explores the effects of varying attention mechanisms and their fusion methods on recognition performance. Random rotation and random occlusion strategies are introduced in the data preprocessing stage to further enhance its ability to recognize local features of the image and improve its stability and reliability in practical applications.

The main contributions of this study are as follows:

1. Based on dairy farm data in a real production environment, a Holstein cowback image dataset under different viewpoints is successfully constructed. This verifies the effectiveness of the method in this paper in a real production environment and provides an important data basis for future related research.
2. A lightweight feature extraction network for Holstein cow individual recognition, CowBackNet, is proposed. By introducing the composite attention mechanism, the network can maintain high recognition accuracy in the case of changes in the shooting angle, enhance the adaptability to external conditions, and ensure the stability and reliability of the model from different angles.
3. Comparing and analyzing with the existing mainstream recognition models, CowBackNet shows significant advantages in recognition efficiency, model size and classification accuracy, especially in the lightweight and computational efficiency of the model, and can provide more efficient reasoning performance and adapt to the deployment requirements in actual production.
4. By introducing gradient-weighted class activation mapping (Grad-CAM), the model’s decision-making process is visualized, significantly improving the interpretability of the individual recognition task. The heatmap analysis visualizes the key regions the model focuses on during the decision-making process, providing a basis for further optimization.

## 2. Materials and Methods

### 2.1. Housing and Laboratory Animals

**Figure 1:**
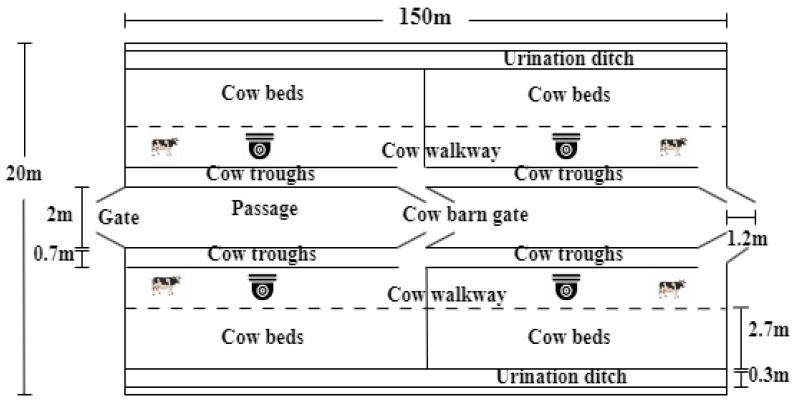
Layout of experimental barn.

The data of this study were collected from 20 May to 10 June 2024. The experimental site was a large commercial dairy farm in Hohhot, Inner Mongolia Autonomous Region. The farm has 5600 dairy cows, including calves and adults, of three breeds: Holstein, Ayrshire and Simmental. The farm has 10 units, each with a length and width of 150 and 20 m, respectively. Each unit has four pens with approximately 90–120 head of cattle per pen. There are two cameras in each pen to collect video data, and we chose pen 2 for breeding black-and-white patterned adult Holstein cows. The layout of the experimental barn is shown in Figure 1.

### 2.2. Data Collection

#### 2.2.1. Video Data Collection

**Figure 2:**
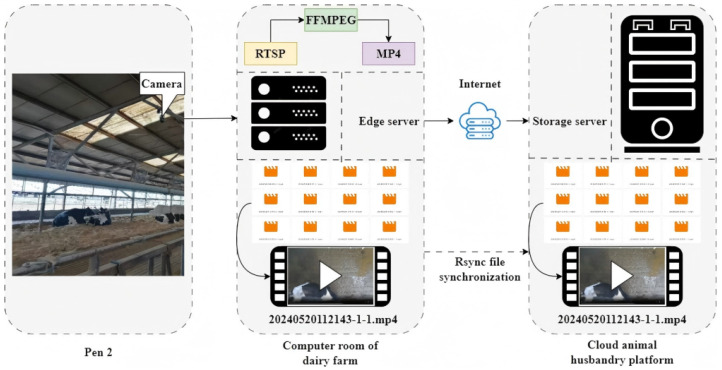
Video data collection.

Data collection for this study was conducted using a DS-2DC4223IW-D dome camera (manufactured by Hikvision, Hangzhou, China), featuring controllable pan–tilt–zoom (PTZ) capabilities with a 360° horizontal and −15° to 90° vertical range of motion. Equipped with 23× optical zoom and 16× digital zoom, and boasting a resolution of 1920 × 1080 pixels, this camera enabled the capture of Holstein cowbacks from diverse viewpoints. Installed atop the barn at a height of 4.3 m, the camera was interfaced with an edge server housed in the farm’s server room. The edge server, comprising a Dell OptiPlex 3070 desktop PC (manufactured by Dell Inc., Round Rock, TX, USA), facilitated real-time video capture via Ethernet using the RTSP protocol from the camera’s visible channel. The captured video streams were instantaneously archived onto the server’s hard disk. Data collection spanned 20 consecutive days, with video files generated every 10 min in MP4 format, adhering to the naming convention “collection start time_camera number.mp4”. This resulted in a comprehensive dataset comprising 2880 video files. The edge server and the cloud animal husbandry platform’s storage server were interconnected via the Internet, facilitating seamless video file transfer to the storage server through the Rsync file synchronization protocol. Post-transfer, the edge server deleted the transferred files to enhance synchronization efficiency and optimize storage space.The data acquisition system employed in this study exhibited robust practical production applicability, demonstrating exceptional end-to-end generalization capabilities and efficient operation within complex farm environments [23]. Figure 2 illustrates the video data collection process.

#### 2.2.2. Data Preprocessing

Since neighboring frames in a video usually have high similarity, directly extracting image features frame by frame may lead to data redundancy, which increases the risk of overfitting the model and limits its generalization ability. Therefore, the video files in this study were processed as follows: the principal activity time of the cows was observed to be 06:00–22:30, and after excluding the video data with no cow activity from the 2880 original videos, the filtered 1980 video files were subjected to frame extraction using FFMPEG software (version 4.3). One frame was extracted every 10 frames to reduce data redundancy while retaining sufficient image data for subsequent analysis. Next, to increase the diversity of the dataset and minimize redundancy, the extracted images were analyzed for similarity using the SSIM algorithm. The parameter was set to 0.78, and finally, the images were cropped and saved using ImageGlass software (version 9.0.11.502). The complete Holstein cowback images were filtered, and the number of Holstein cow images under each ID was not less than 40, ensuring that the images of each cow were diverse enough to support subsequent model training and accurate recognition. After the above steps, the final selected dataset includes back images of 22 cows with different viewpoints. To better simulate the production environment and improve the model’s generalization ability, we performed random image enhancement when loading the model training data, including horizontal or vertical flipping, adding noise, and small object occlusion simulating the distribution of different orientations, visual interference, and lens pollution caused by changes in light conditions that are common in actual production.

#### 2.2.3. Dataset Construction

**Figure 3:**
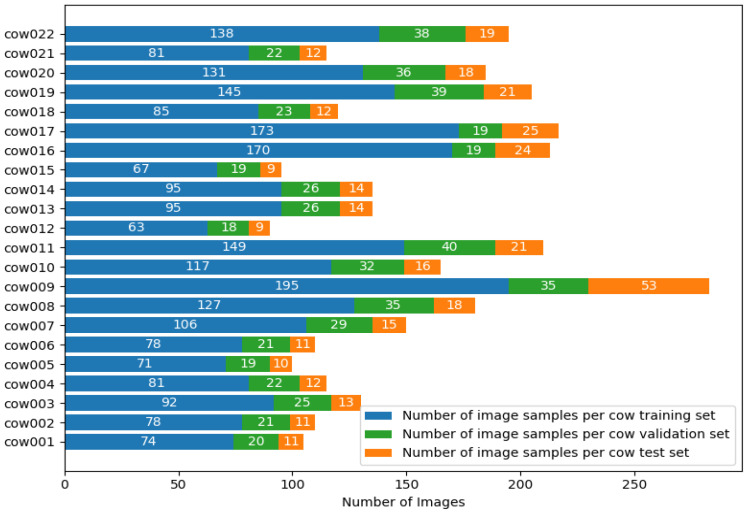
Distribution of sample counts per Holstein cow across the training, validation, and testing subsets of the CowBack dataset.

In this paper, two datasets were constructed, the first of which was collected in pen 2. A total of 3410 Holstein cowback images were obtained after data preprocessing, named the CowBack dataset, and divided into training set, validation set, and test set in a ratio of 7:2:1. To facilitate back image processing in the later recognition stage, the back images included part of the neck. Figure 3 displays the distribution of sample counts per Holstein cow across the training, validation, and testing subsets of the CowBack dataset.

**Figure 4:**
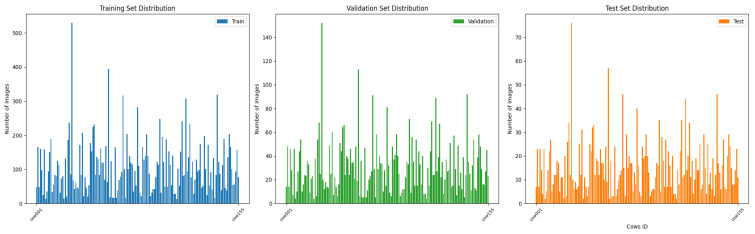
Distribution of sample counts per Holstein cow across the training, validation, and testing datasets of the Cows2021 dataset.

**Figure 5:**
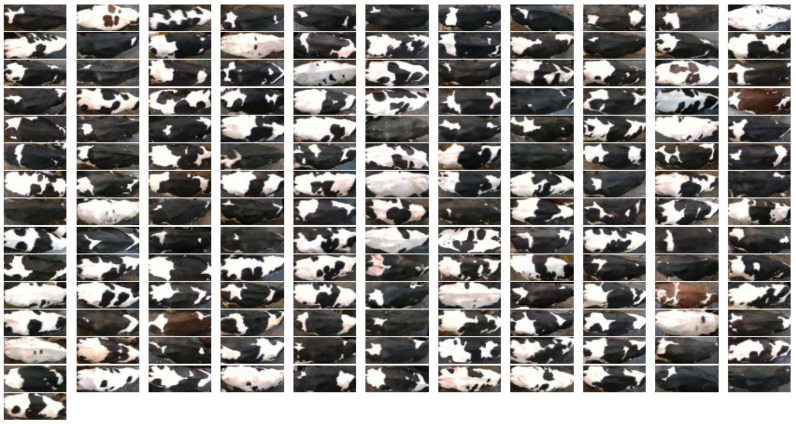
Back sample of each Holstein cow in the Cows2021 dataset.

Furthermore, to comprehensively assess model performance, we incorporated the RGB image dataset Cows2021 [24] as a benchmark dataset, encompassing varied application contexts and diverse feature representations. It includes 186 Holstein Friesian cows obtained through the Inter D435 at the University of Bristol’s Windhurst Farm, Village of Langford, UK. The camera was pointed downward from 4 m above ground level to the walkway between the milking parlor and the fence. The dataset was parsed to 1280 × 720 pixels per frame, 8 bits per RGB channel. It contains 10,402 still images and 301 videos (each 5.5 s long) at a frame rate of 30 fps. Although the Cows2021 dataset contains raw individual cow image data, it includes a few samples of some individuals. After data sorting and screening, a Cows2021 dataset containing 155 Holstein cows with 23,918 back images was finally constructed. Figure 4 illustrates the distribution of sample counts per Holstein cow across the training, validation, and testing datasets, while Figure 5 depicts the back sample of each Holstein cow.

Furthermore, the Cows2021_mix dataset was constructed, which includes both Holstein cowback images in top view, i.e., part of the Cows2021 dataset, and Holstein cowback images in different viewpoints, i.e., the CowBack dataset. Simulating data variability in real environments by fusing images from different viewpoints helps to train recognition models that can effectively respond to a variety of scenarios while ensuring that the number of Holstein cows is consistent with the number of Holstein cows in the Cows2021 dataset to ensure the stability of the experimental conditions and the balance of the data. Finally, 24,592 images containing 155 Holstein cows were obtained. An overview of the above-constructed dataset is shown in Table 1.

### 2.3. Methods

**Figure 6:**
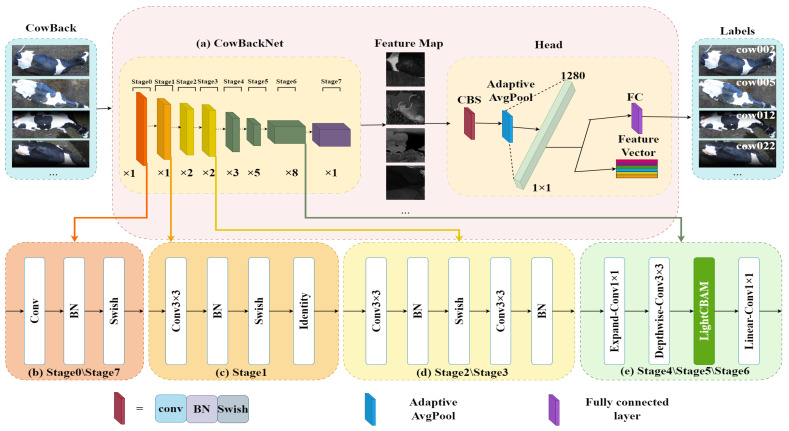
Framework.

This study aims to obtain Holstein cowback images for Holstein cow individual recognition from different viewpoints. The proposed individual recognition framework diagram is shown in Figure 6. In this study, we designed and implemented a lightweight feature extraction network called CowBackNet, which accepts an image of the back of a Holstein cow as input and outputs a 7 × 7 pixel feature map. These feature maps are then passed to a module containing convolution, batch normalization, Swish activation functions (collectively referred to as the CBS layer), and an adaptive mean pooling layer. The aforementioned processing sequence comprises the head module, tasked with transforming high-dimensional feature maps into a compact 1280-dimensional feature representation. Subsequently, this representation is input into a fully connected layer, whose function is to identify 22 distinct Holstein cows, producing the definitive classification result.

Several evaluation metrics verified the effectiveness of the present framework, including the accuracy change profile during training, the accuracy during the testing phase, and the number of parameters and FLOPs analyzed for the model. These indicators collectively measure the model’s performance and efficiency in recognizing individual Holstein cows.

#### 2.3.1. CowBackNet Feature Extraction Module

Deep separable convolution (DSC) [25] is known to drastically decrease the parameter count in convolutional layers. By integrating DSC with inverse residual structures [26], a lightweight model can attain superior performance. To achieve individual cow recognition by extracting texture information from the cow’s back necessitates a high degree of detail in feature extraction from the imagery, posing a significant challenge in designing an efficient lightweight feature extraction module. Among them, the EfficientNetV2 network [27] is improved and proposed based on the EffcientNet network [28]. Combining DSC and attention mechanisms mainly solves the problems of slow training of large-size images and low efficiency of deep convolution for shallow networks. It introduces the Fused-MBConv structure to simplify the convolution operation, speed up the training, and optimize the network structure with Neural Network Architecture Search (NAS). In addition, the last parameter-intensive stage is removed, reducing the number of parameters and memory consumption of the model. These improvements allow EfficientNetV2 to increase training efficiency and minimize model complexity while maintaining high accuracy.

The EfficientNetV2 network uses the SE (squeeze-and-excitation) attention mechanism [29]. However, the SE mechanism only focuses on the channel information and ignores the spatial information, which may be insufficient to deal with complex backgrounds or variable cow postures. CBAM (Convolutional Block Attention Module) [30] is a comprehensive attention mechanism proposed in 2018 to consider the relationship between space and channel simultaneously to enhance the feature extraction capability of the network. CBAM focuses attention on the channel and spatial dimensions sequentially, and this step-by-step processing allows CBAM to accurately reshape the feature maps and optimize the representation of the features, thus improving the model performance. However, CBAM involves attentional processing for both channel and spatial dimensions, which may increase the computational burden, especially in resource-constrained environments. ECA (Efficient Channel Attention) [31] is a lightweight attentional mechanism focusing on the channel dimension, proposed in 2020. Unlike traditional SE mechanisms that require complex spatial operations (e.g., global average pooling followed by a fully connected layer), ECA omits complex dimensional transformations and the problem of learning too many parameters by using a one-dimensional convolution to apply attention directly to the channel dimension. However, since ECA mainly focuses on adjusting the channel dimension, it may ignore the spatial relationship in the image, which may be insufficient for capturing the subtle changes in the cow’s back. The coordinate attention (CA) mechanism [32] is a new lightweight, plug-and-play attention mechanism proposed in 2021, which can target both spatial and channel dimensions with more comprehensive feature-capturing capability. It can significantly enhance the model’s representation of the input data by emphasizing important features and suppressing minor ones. Although the CA is designed to be more comprehensive, its performance may still be limited by the model’s ability to respond to specific spatial features under extreme or uncommon pose and lighting conditions.

**Figure 7:**
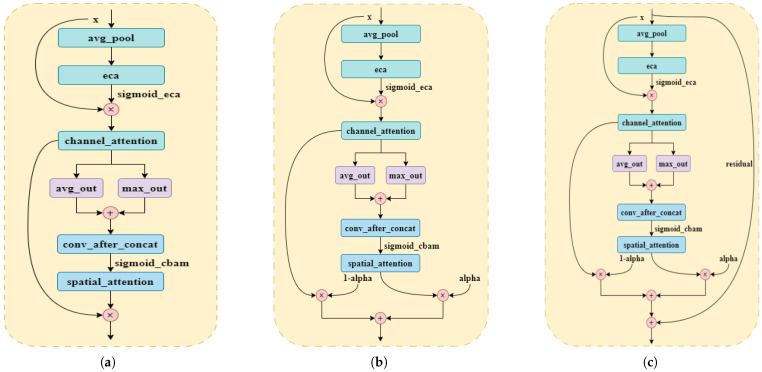
Multi-attention fusion mechanism approaches: (**a**) Tandem fusion. (**b**) Weighted fusion. (**c**) Weighted fusion and introduction of residual connections.

Completing individual cow recognition through cowback images is part of the fine-grained image recognition task, which requires the model to be able to capture minor individual differences, and any attention mechanism that fails to enhance the region associated with individual features may affect the recognition accuracy, which is a generic problem of the above attention mechanisms. ECA focuses on efficiently utilizing global information to adjust the weights of the channels. In contrast, CBAM further integrates spatial attention on this basis, which allows it to parse which regions in the image are more critical in a more detailed way. Therefore, this paper aims to integrate these two attention mechanisms to capture multi-level and multi-angle features from global to local and enhance the model’s ability to understand image details. At the same time, the applicability and resource efficiency of different attention mechanisms on specific tasks are considered to find the optimal balance. The multi-attention fusion mechanism approach is shown in Figure 7.

This study focuses on three types of fusion, namely tandem fusion, weighted fusion, and weighted fusion with the introduction of residual connectivity to take advantage of the complementary strengths of the different types of attentional mechanisms, to enhance the model’s comprehension and characterization of the input data. As shown in Figure 7a, ECA is first implemented using global average pooling and a one-dimensional convolution. The channel attention maps generated in this step multiply the original inputs element-wise to produce an adjusted feature map. Next, spatial attention processing is performed on the channel attention-adjusted feature maps obtained in the previous step. Specifically, the mean and maximum values are first computed separately from the feature map, the two images are stitched along the channel direction, and then a spatial attention map is generated using a convolutional layer and a sigmoid activation function. Again, this spatial attention map creates the final output by element-wise multiplication of the channel attention-adjusted feature maps. ECA can still effectively capture important channel information by reducing the model parameters and maintaining computational efficiency; the spatial attention of CBAM further strengthens the identification of key spatial features. By combining the spatial attention of ECA and CBAM, the model can be more flexible in adapting to essential features and improve overall recognition accuracy. Although ECA is designed to reduce FLOPs, the whole fusion module still adds extra computational steps, especially the convolution operation in the spatial attention module, which may increase the overall computational burden of the model, affecting the responsiveness and utility of the model.

When applying Figure 7a to cowback individual recognition, a balance must be found between enhancing the feature recognition capability and maintaining the model’s efficiency. Figure 7b attempts to address this issue by introducing the Alpha parameter, where the model linearly interpolates between the feature maps from the channel attention processing and the results from the spatial attention processing, thus balancing the contributions of the two attention mechanisms. This fusion provides greater flexibility and adaptability, enabling the model to automatically learn the optimal weights of the attention mechanisms based on the training data, and thus can improve the recognition accuracy of cowback individuals by emphasizing key features and regions. Although introducing the Alpha parameter provides flexibility, it also increases the difficulty of model tuning and requires detailed experiments to determine the optimal values.

To cope with the problems of the first two fusion approaches, a third fusion strategy is proposed in this study (Figure 7c). This strategy improves the robustness of the model in complex backgrounds, especially the ability to adapt to the cow’s back image in different viewpoints, by introducing learnable Alpha parameters and residual connections. Precisely, by introducing the learnable Alpha parameter, the weight ratio between channel and spatial attention is dynamically adjusted, enabling the model to maintain a low computational burden while improving performance. In addition, the introduction of residual connection effectively solves the problem of information loss in the deep network, which makes the network maintain stability when increasing the depth and reduces the training difficulty, thus further improving the model’s expressive ability and generalization ability and avoiding the risk of overfitting. The fusion strategy enhances the model’s ability to recognize cowback images with different shooting angles and background complexity by flexibly adjusting the contribution ratio of other modules and significantly improves its adaptability and efficiency in complex environments. We named the module LightCBAM and placed it in Figure 6e as an effective solution to recognize individual Holstein cows under different viewpoints.

#### 2.3.2. CowBackNet Network Structure

A novel, efficient network for cow pattern feature extraction, termed CowBackNet, was devised utilizing the designed LightCBAM module. Table 2 details its intricate structure, segmented into distinct stages ranging from 0 to 7. Each stage specifies the operational module employed and the corresponding input image resolution. A standard convolutional layer is first used for the initial feature extraction of the image. The expansion from three RGB channels in the input layer to 32 channels increases the features’ dimensionality and helps capture more edge and texture information. An enhanced version of the convolution module is then used, keeping the resolution constant, to continue extracting deeper features. Then, an edge enhancement module using residual linking is applied to capture the edge information of the image, which helps pinpoint the contours and features of the cow’s back. Different step sizes and resolution strategies are used in the two sub-layers to achieve practical feature preservation and spatial compression. The inverse residual structure is used in these layers, and the LightCBAM module is introduced to increase the network depth and complexity at a lower parameter cost. The fine processing of the feature maps at different stages is realized by precisely controlling the resolution and the step size to gradually reduce the size of the feature maps to improve computational efficiency. The final two layers integrate and analyze extensive feature data, bolstering the model’s recognition capabilities. Subsequently, an average pooling technique refines the high-level semantic features, yielding a 1280D feature vector as output. This network architecture is beneficial in accomplishing the task of individual cow recognition through cowback images by analyzing the image features layer by layer in depth and combining the residual connectivity and attention mechanisms; it effectively improves the feature characterization ability and recognition accuracy, as well as ensures the computational efficiency of the network.

### 2.4. Evaluation Indicators

In this study, the primary performance indicators for assessing the classification model are the top-1 and top-5 accuracy rates, offering insights into the model’s precision in cow individual identification tasks. Top-1 accuracy is used to measure whether the highest probability category predicted by the model matches the true category and is calculated as shown in Equation (1):

(1) $$Top\text{-}1\;\mathrm{Accuracy}=\frac{N_{\mathrm{current}}}{N_{\mathrm{total}}}$$

However, top-5 accuracy measures whether the proper category is included in the top 5 highest probability categories predicted by the model and is calculated as described in Equation (2):

(2) $$Top\text{-}5\;\mathrm{Accuracy}=\frac{N_{top\text{-}5\;\mathrm{current}}}{N_{\mathrm{total}}}$$

where $N_{\mathrm{total}}$ signifies the aggregate count of samples within the test dataset, indicating the number of benchmarks for model evaluation. $N_{\mathrm{current}}$ refers to the number of correct predictions in top-1 accuracy, indicating the number of samples in which the first prediction is accurate. $N_{top\text{-}5\;\mathrm{current}}$ refers to the number of correct predictions in top-5 accuracy, indicating the number of samples in which the first five predictions have proper labels.

In addition, the number of parameters, the total number of parameters to be trained in the model training, is used to measure the model’s size. FLOPs [33], the number of floating point operations, is used to measure the complexity of the algorithms and is often used as an indirect measure of the speed of the neural network model. The calculation is shown in Equation (3):

(3) $$\mathrm{FLOPs}=2\mathrm{hw}\;\times \;(c_{\mathrm{in}}\;\times \;K^{2}+1)\;\times \;c_{\mathrm{out}}$$

where h, w, and $c_{\mathrm{in}}$ denote the height, width, and number of channels of the input feature map, and $c_{\mathrm{out}}$ denotes the number of channels of the output feature map, respectively. K represents the width of the convolution kernel.

## 3. Results

### 3.1. Experimental Setup and Parameters

The experimental platform in this study comprises a Linux server running the Ubuntu Server 22.04 operating system. The server’s hardware specifications feature two Intel Xeon Gold 6139M processors clocked @ 2.30 GHz, complemented by 128 GB of RAM and eight NVIDIA GeForce RTX 3090 GPUs. The software environment includes Python version 3.8.19, CUDA 11.3, and a suite of deep learning frameworks: PyTorch 1.10.1, MMEngine 0.10.4, and MMPretrain 1.2.0. Table 3 outlines the study’s learning algorithms, optimized using the Adam optimizer with an initial learning rate of 0.0005, a weight decay parameter of 0.0001, and a batch size set to 32.

### 3.2. Selection of Feature Extraction Networks

**Figure 8:**
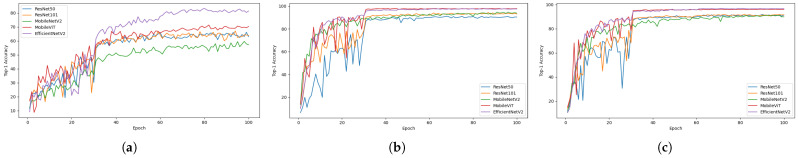
Changes in accuracy during the training phase of the model: (**a**) CowBack. (**b**) Cows2021. (**c**) Cows2021_mix.

This study evaluated ResNet50, ResNet101, MobileNetV2, MobileViT, and EfficientNetV2 as feature extraction networks, comparing their test set performance comprehensively. The results are detailed in Table 4. In our self-built CowBack test set of 22 Holstein cows with back images acquired from different viewpoints for individual recognition, EfficientNetV2 achieves 76.61% accuracy, and the other models perform lower than EfficientNetV2. During the individual identification of 155 Holstein cows in the Cows2021 dataset, which consists of back images captured from a top–down perspective, EfficientNetV2 exhibited superior performance, achieving a top-1 accuracy rate of 95.69% and a top-5 accuracy rate of 98.76%. ResNet50 performs the worst but achieves 91.46% top-1 accuracy and 96.78% top-5 accuracy. MobileNetV2 and MobileViT performed slightly lower than EfficientNetV2. Also, in the Cows2021_mix test set of hybrid multiview, the individual recognition of 155 Holstein cows still had the highest recognition accuracy with EfficientNetV2, with a top-1 accuracy of 93.56% and top-5 accuracy of 97.70%. It shows that the EfficientNetV2 model can still perform optimally even when mixing the back image data of Holstein cows in a real production environment. Based on the top-1 and top-5 accuracy results across the three datasets, EfficientNetV2 was chosen as the foundational network for feature extraction in Holstein cow individual identification in this research. Figure 8 illustrates the evolution of top-1 accuracy during the training phase of each base model.

### 3.3. Single-Attention Mechanism

To validate the effectiveness of the selected attention mechanisms, this study compares multiple-attention mechanisms on the CowBack, Cows2021 and Cows2021_mix datasets to evaluate their impact on classification performance. These attention mechanisms include CA, CBAM and ECA, as shown in Table 5.

The experimental results show that after replacing the original SE attention mechanism in the EfficientNetV2 model, the CA, CBAM, and ECA models all showed performance improvement in identifying individual cows. CBAM and ECA improved the accuracy of top-1 identification on the CowBack dataset by 7.5% and 11.17%, respectively.

### 3.4. Multi-Attention Fusion Mechanism

This section combines the advantages of both CBAM and ECA attentional mechanisms. Specifically, it analyzes three attentional fusion strategies: tandem fusion, weighted fusion, and weighted fusion combined with residual joining (as shown in Table 6). The effects of these fusion strategies on the recognition performance are evaluated through experiments on three datasets: CowBack, Cows2021, and Cows2021_mix.

The experimental results show that the recognition results using weighted fusion and introducing residual connectivity outperform those using a single-attention mechanism on all the test datasets from different viewpoints. This fusion approach not only enhances the model’s focus on channel information but also overcomes the shortcomings of the SE attention mechanism that ignores spatial information, especially when dealing with real environmental images derived from complex backgrounds and cows with variable postures in the CowBack dataset, which show significant improvements. This fusion approach improves the top-1 recognition accuracy by 0.52% over using only the ECA attention mechanism.

### 3.5. Model Performance Analysis

**Figure 9:**
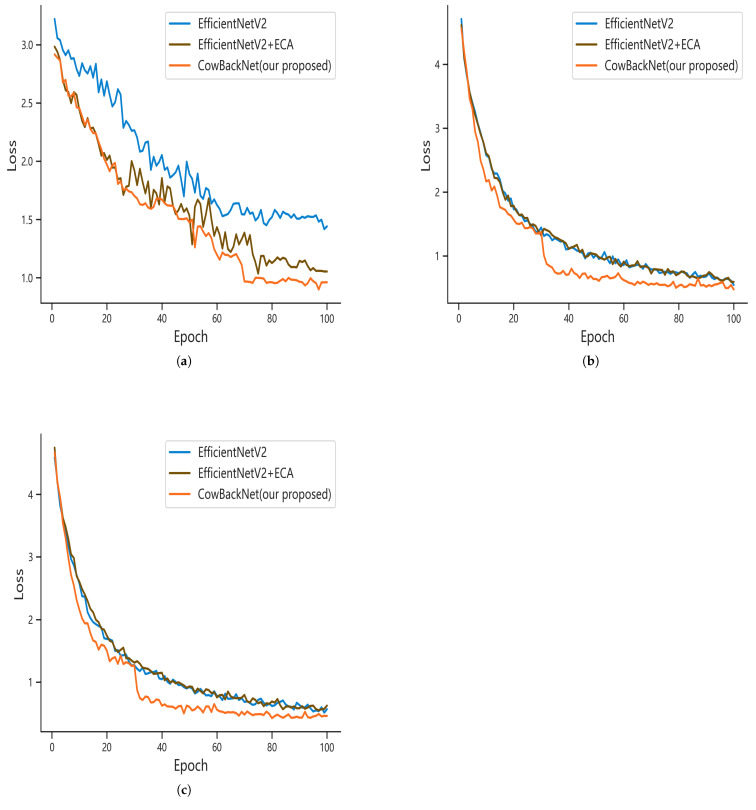
CowBackNet model vs. benchmark model loss curves for different datasets: (**a**) CowBack. (**b**) Cows2021. (**c**) Cows2021_mix.

To further evaluate the performance of CowBackNet, we added two benchmark models for comparison, i.e., EfficientNetV2 and EfficientNetV2 + ECA. The relationship between the loss values, the number of model iterations, and the corresponding curves during the training process are shown in Figure 9. Figure 9 shows that after 100 iterations, the model tends to converge. Finally, the loss of the CowBackNet model is stabilized at around 0.951, 0.457, and 0.54 under the CowBack, Cows2021, and Cows2021_mix datasets, respectively, which proves that the improved model has a strong learning ability.

Then, we compared CowBackNet with mainstream recognition models, including ResNet, MobileNet, MobileViT, and EfficientNetV2. In addition, it was also compared with each model of EfficientNetV2 with the introduction of a single-attention mechanism. Table 7 shows the statistical results of the proposed CowBackNet with mainstream recognition models. As can be seen from Table 7, compared with ResNet50 and ResNet101, which also have a residual network structure, the FLOPs and the number of parameters of CowBackNet are significantly reduced. Compared with the lightweight models MobileNet, MobileViT, and EfficientNetV2, although the number of parameters is improved, CowBackNet achieves a higher accuracy rate. In addition, the top-1 accuracy of CowBackNet is 11.69%, 4.93%, 4.19%, and 0.52% higher than that of EfficientNetV2, EfficientNetV2 + CA, EfficientNetV2 + CBAM, and EfficientNetV2 + ECA, respectively. In terms of computation, the FLOPs of CowBackNet are all lower than those of EfficientNetV2, EfficientNetV2 + CA, EfficientNetV2 + CBAM, and EfficientNetV2 + ECA, and in terms of model sizes, CowBackNet outperforms the EfficientNetV2, EfficientNetV2 + CA, EfficientNetV2 + CBAM, and EfficientNetV2 + ECA, reaching 6.096 MB. It is shown that the complexity of CowBackNet is significantly lower than the above model.

**Figure 10:**
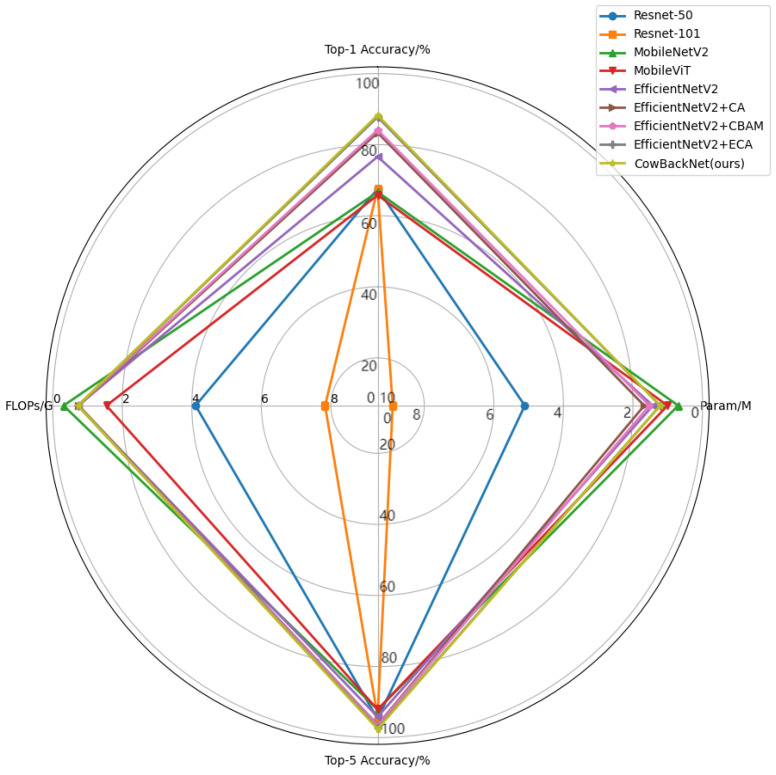
Comparison of FLOPs, number of parameters, and accuracy of each model.

The data in Table 7 were analyzed using a radar chart, and the results are shown in Figure 10. Based on the above results and analysis, the proposed CowBackNet model has certain advantages in terms of accuracy, model size, and efficiency compared with mainstream classification models for the task of individual cow recognition.

### 3.6. Model Interpretability Analysis

To fully evaluate the performance of CowBackNet for Holstein cow individual recognition in a realistic environment, we generated heatmap visualization results using gradient-weighted class activation mapping (Grad-CAM) [34], which are shown in Table 8. This is the result of a visualization of individual identification through the back of a Holstein cow. All Holstein cow samples in Table 8 are from the test set of the CowBack dataset, where Holstein cowback samples with salient and weak features are randomly selected and salient features refer to features easily recognized and understood in an image. Examples of salient features include noticeable color patches, specific markings or labels, etc. Weak features refer to those image features that are less obvious or easily overlooked, which may not directly affect the interpretation and recognition of the image as much as the salient features, but still have recognition value in some cases—for example, subtle hair textures, slight color gradients, etc. In addition to visualizing the recognition results on the original image, the recognition results are also visualized on the image after random rotation, random occlusion, and different viewpoints, which are used to verify that the model is still able to focus on the same or similar regions of interest after the above operations. In addition, the test set of the Cows2021 dataset of Holstein cowback images acquired from the top-view angle was also used to visualize the recognition results of individual Holstein cows, as shown in Table 9. In this case, the warm color in the heatmap indicates that the model pays excellent attention to the region, i.e., the model believes that the area makes a more significant contribution to the decision.

When using EfficientNetV2 to recognize individual Holstein cows in a realistic environment, it was susceptible to background interference, resulting in less attention being paid to the back region of the Holstein cow. This is the main reason why the accuracy of the EfficientNetV2 model in the CowBack dataset for Holstein cow individual recognition is lower than the recognition accuracy of the Cows2021 dataset constructed from the top view. However, by optimizing the operation of EfficientNetV2, as shown in the heatmap of CowBackNet, after visualizing the recognition results of the original image of the same model, even in the face of the interference of transformation or noise, the results of the image visualized using Grad-CAM did not change much from the visualization results of the original image, and the model was still able to capture the key information accurately, indicating that the model has better generalization ability and anti-interference ability; on the other hand, after visualizing the recognition results of the images after the same operation with different models, the warmer color is darker, indicating that the CowBackNet model displays greater attention in the critical feature regions, and can make a decision from the practical features, focusing on the key areas that have decision-making impact on the classification, which will, in turn, affect the recognition accuracy.

This result confirms the effectiveness of the optimization operation, showing that CowBackNet can enhance the focus on the back region of Holstein cows while mitigating the interference of the background. In addition, it is confirmed that the model significantly enhances its context-awareness capability by introducing a multi-attention fusion mechanism, which enables the model to more accurately localize and focus on key features on the back of Holstein cows when processing image data. Even when the shooting angle changes, the introduction of LightCBAM enables the model to automatically adjust the channel and spatial attentional mechanisms, which allows the model to more accurately extract the features on the back of the cow under different viewpoints and enhances its ability to recognize individual cows. In conclusion, this approach further optimizes the feature extraction process by effectively filtering and suppressing irrelevant background noise, thus improving the robustness and accuracy of the overall model. The heatmap visualization evaluation results show that these optimization operations help improve individual Holstein cows’ recognition performance in a completely realistic environment.

## 4. Discussion

In this study, our proposed CowBackNet model achieves 88.30%, 95.86%, and 94.32% top-1 accuracy on the CowBack, Cows2021, and Cows2021_mix test sets, respectively. As can be seen from Table 4 and Table 6, compared to the EfficientNetV2 model, the CowBackNet model improves the top-1 accuracy by 0.17% and 0.76% on the Cows2021 and Cows2021_mix test sets, respectively, while the improvement is significant up to 11.69% on the CowBack test set. This significant performance improvement highlights that the CowBackNet model has a stronger generalization ability in processing Holstein cowback images in a completely realistic environment. In addition to accuracy, the model’s computational efficiency and storage requirements are also key factors that must be considered in practical applications. Among them, as shown in Table 7, the FLOPs of the CowBackNet model reach 0.727 G, indicating that its computational demand in inference is moderate, making the model suitable for resource-constrained environments. In addition, the model’s overall size is only 6.096 MB, which further reduces storage pressure and improves deployment flexibility. This combination reduces the running cost and helps the model be used in mobile devices and embedded systems, enabling the CowBackNet model to perform efficient individual identification of Holstein cows in real-time scenarios. As a result, the CowBackNet model optimizes resource usage efficiency while maintaining high accuracy, demonstrating the potential for application in modern Holstein dairy cow management systems.

**Figure 11:**
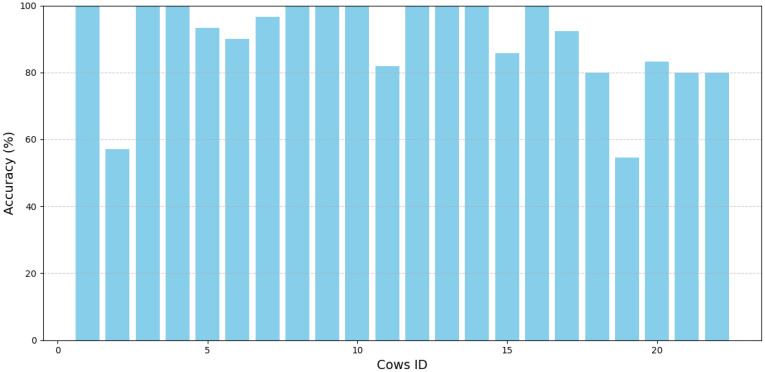
Recognition accuracy per cow for CowBack dataset.

Furthermore, the CowBackNet model has demonstrated significant performance in the task of Holstein cow individual recognition in multiple viewpoints, accurately recognizing Holstein cow individuals in the vast majority of cases. As shown in Figure 11, cow001, cow003, cpw004, cow008, cow009, cow010, cow012, cow013, cow014, and cow016 all have 100% recognition accuracy, and cow005, cow006, cow007, cow011, cow015, cow017, cow018, cow020, cow021, and cow022 can achieve more than 80% recognition accuracy, of which cow002 and cow019 have the lowest recognition accuracy, mainly due to the fact that the back image features of these two cows are relatively few, causing the model to face difficulties in recognition. However, despite the model’s excellent performance across multiple viewpoints, we still observed that recognition misalignment occurred in a few specific viewpoints. Some examples of misrecognition in the CowBack test set are shown in Table 10. Due to the change in shooting angle, some images have viewpoint-related feature loss (e.g., cow006, cow011), which leads to incomplete essential features in the image, affecting the feature extraction process and thus leading to misrecognition. In addition, background interference is particularly prominent in large-scale farm environments, especially in the presence of other Holstein cows (e.g., cow005, cow015). The complexity of the background and the presence of other Holstein cows may lead to difficulties for the model in distinguishing the unique features on the backs of different individual Holstein cows, which may affect the recognition accuracy. Rapid movement of Holstein cows is also an essential factor leading to recognition errors. The motion blur produced by fast walking (e.g., cow007, cow022) results in the loss of detailed information in the image, leading to the inability to accurately capture the key features of the Holstein cow’s back, further affecting the model’s recognition ability.

**Figure 12:**
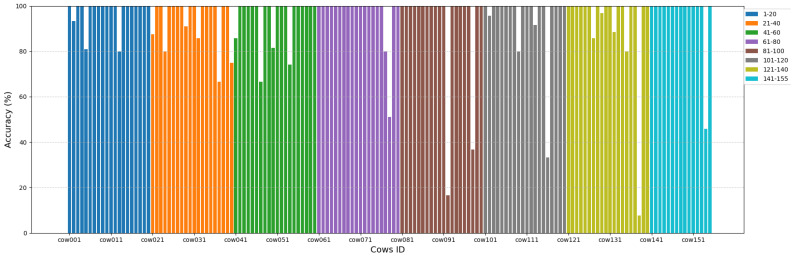
Recognition accuracy per cow for Cows2021 dataset.

As shown in Figure 12, recognition accuracy of cow037, cow040, cow047, cow054, and cow78 is below 80%, and individual ones such as recognition accuracy of cow092, cow098, cow116, and cow138 can only reach about 20–40%, while the rest can reach about 80–100%. However, despite the excellent performance of the CowBackNet model in image processing for the top view, we still observed some misrecognized samples in the Cows2021 test set. Table 11 shows some examples of misrecognition samples in the Cows2021 test set. When analyzing these misrecognition samples, we observe that issues such as low texture complexity, image blurring, information loss, background interference, and pose changes impact the model’s recognition precision directly. In particular, low texture complexity refers to the lack of significant texture variations in an image, which leaves less valid information, making it difficult to extract detailed features. Due to the extensive black-colored areas on the backs of Holstein cows, their distinctive features are predominantly found in the white-patterned regions. When the size of the white areas on different Holstein cows is limited, as exemplified by images such as cow050, cow109, and cow154, the subtle variations in these white patterns among individuals pose significant challenges in accurately distinguishing one Holstein cow from another, thereby intensifying the model’s recognition difficulty. In addition, as the movement of Holstein cows leads to image blurring, e.g., in the images of cow001 and cow138, the different parts of the image are too complex to be clearly distinguished, and sufficient helpful information cannot be extracted, so the practical information of the image is significantly reduced, which ultimately leads to recognition errors. In addition, information loss is another major factor leading to recognition errors. Images of cow005, cow077, and cow092 have different degrees of information loss, leading to incomplete feature extraction. This information loss seriously interfered with the model’s accurate recognition of individual features of Holstein cows, which in turn affected the model’s recognition rate. Background interference (cow054) and pose variation (cow116) in the images are also key factors for misrecognition.

In summary, textural features [35] on the back of Holstein cows vary from breed to breed and pose different challenges regarding individual recognition. While focusing on the back images of Holstein cows with salient and weak features at the same time, the diversity of the training data is enhanced by random rotation, random occlusion and other methods to help the model learn more global features and detailed features, and to improve its recognition ability on weak feature images with solid colors or no obvious patterns as much as possible.

However, due to the lack of strong texture features and low image contrast in weak feature images, the effect of feature extraction using the model proposed in this paper is still not as good as that of significant feature images. Future research can consider combining multimodal information, such as near-infrared images, temperature data, or depth images, and combining these with visual images to form multimodal inputs to make up for the deficiencies of pure color images and to help the model differentiate better through additional channel information. Alternatively, a transfer learning approach can apply deep networks pre-trained in other tasks (e.g., face recognition, object detection) to the cow recognition task to learn more generalized features to help improve the recognition of weak feature images that are difficult to distinguish. However, due to time and resource constraints, we were not able to delve deeper into this topic in the current study. The main considerations are that integrating multimodal information faces several challenges and limitations: first, the data acquisition process of different modalities is complicated and requires additional equipment and technical support, which poses challenges in terms of time and cost. Second, effective integration of data from different modalities requires complex algorithms and model design to ensure complementarity and consistency of information, which is technically challenging. Third, multimodal inputs may require larger training datasets and longer training times so that models can learn effective features, which may be difficult to achieve with limited resources. In addition, since Holstein cowback images are obtained from pen 2, although adult Holstein cowback images are generated by taking into account the various situations in the production environment as much as possible, they may not cover all environmental conditions, thus affecting the accuracy. Subsequent studies could consider collecting images at different dairy farms and evaluating the effects caused by indoor and outdoor lighting environments to improve the individual cow recognition model using a more diverse dataset. Future work will aim to extend the dataset and optimize the model structure to enhance the model’s usefulness in natural production environments and address the challenges of applications in this setting.

## 5. Conclusions

In this research, we introduced an enhanced convolutional neural network design for identifying individual Holstein cows, which boosts the model’s capacity to discern Holstein cows across various orientations, perspectives, and partial obscurations through the integration of spatial- and channel-wise feature extraction methodologies. Among them, we proposed the CowBackNet feature extraction network and explored the effects of single-attention and multi-attention fusion mechanism strategies on the recognition effect. This model achieves an 88.30% accuracy on the CowBack dataset constructed with different viewpoints, a 95.85% recognition accuracy on the Cows2021 dataset with top viewpoints, and a 94.32% on the Cows2021_mix dataset constructed by fusing different viewpoints. This work provides an effective solution for Holstein cow individual recognition at the technical level and contributes valuable technical references for subsequent research in related fields.

## Figures and Tables

**Table 1:** An overview of the dataset.

Datasets	Total Number of Cows	Total Images of Cows			
		Train	Val	Test	Total
Cows2021	155	16,684	4820	2414	23,918
CowBack	22	2411	657	342	3410
Cows2021_mix	155	17,158	4951	2483	24,592

**Table 2:** An overview of the dataset.

Stage	Operator	Kernel Size	Resolution	Stride	Channels	Layers
0	ConvModule	(3, 3)	(224, 224)	(2, 2)	32	1
1	EnhancedConvModule	(3, 3)	(112, 112)	(1, 1)	16	1
2	EdgeResidual	[(3, 3), (1, 1)]	(112, 112)	[(2, 2), (1, 1)]	[64, 32]	2
3	EdgeResidual	[(3, 3), (1, 1)]	(56, 56)	[(2, 2), (1, 1)]	[128, 48]	2
4	MyInvertedResidual	[(1, 1), (3, 3), (1, 1)]	(28, 28)	[(1, 1), (2, 2), (1, 1)]	[192, 192, 96]	3
5	MyInvertedResidual	[(1, 1), (3, 3), (1, 1)]	(14, 14)	[(1, 1), (1, 1), (1, 1)]	[576, 576, 112]	5
6	MyInvertedResidual	[(1, 1), (3, 3), (1, 1)]	(7, 7)	[(1, 1), (2, 2), (1, 1)]	[672, 672, 192]	8
7	ConvModule	(1, 1)	(7, 7)	(1, 1)	1280	1

**Table 3:** Experimental parameter setting.

Parameters	Value
Optimizer	Adam
Learning rate	0.0005
Weight decay	0.0001
Batch size	32
Learning rate scheduler	Cosine Annealing Strategy

**Table 4:** Comparison of results for different base models.

Datasets	Models	Top-1 Accuracy (%)	Top-5 Accuracy (%)
CowBack	Resnet-50	67.54	94.74
	Resnet-101	67.54	94.15
	MobileNetV2	66.67	92.11
	MobileViT	65.79	92.11
	**EfficientNetV2**	**76.61**	**94.15**
Cows2021	Resnet-50	91.46	96.78
	Resnet-101	91.50	96.90
	MobileNetV2	91.67	96.73
	MobileViT	93.79	97.27
	**EfficientNetV2**	**95.69**	**98.76**
Cows2021_mix	Resnet-50	89.64	96.40
	Resnet-101	90.55	96.40
	MobileNetV2	90.05	96.42
	MobileViT	92.43	97.38
	**EfficientNetV2**	**93.56**	**97.70**

**Table 5:** Comparison of the results for the different attention mechanism methods.

Datasets	Attention	Top-1 Accuracy (%)	Top-5 Accuracy (%)
CowBack	CA	83.37	96.40
	CBAM	84.11	96.60
	**ECA**	**87.78**	**97.31**
Cows2021	CA	95.05	97.98
	CBAM	95.26	98.20
	**ECA**	**96.29**	**98.64**
Cows2021_mix	CA	91.52	97.70
	CBAM	92.19	97.85
	**ECA**	**93.19**	**97.99**

**Table 6:** Comparison of the results for the different attention fusion methods.

Datasets	Fusion Approach	Top-1 Accuracy (%)	Top-5 Accuracy (%)
CowBack	Tandem Fusion	82.40	96.82
	Weighted Fusion	85.33	97.07
	**Weighted Fusion + Residual Connectivity**	**88.30**	**97.56**
Cows2021	Tandem Fusion	95.19	98.02
	Weighted Fusion	95.01	98.41
	**Weighted Fusion + Residual Connectivity**	**95.86**	**98.50**
Cows2021_mix	Tandem Fusion	90.90	96.98
	Weighted Fusion	92.83	97.70
	**Weighted Fusion + Residual Connectivity**	**94.32**	**98.10**

**Table 7:** Comparison of the results for different mainstream recognition models.

Models	FLOPs (G)	Param (M)	Top-1 Accuracy (%)	Top-5 Accuracy (%)
Resnet-50	4.109	25.557	67.54	94.74
Resnet-101	7.832	44.549	67.54	94.15
MobileNetV2	0.313	3.505	66.67	92.11
MobileViT	1.546	5.037	65.79	92.11
EfficientNetV2	0.728	7.140	76.61	94.15
EfficientNetV2+CA	0.751	8.386	83.37	96.40
EfficientNetV2+CBAM	0.729	7.637	84.11	96.60
EfficientNetV2+ECA	0.727	6.095	87.78	97.31
**CowBackNet (ours)**	**0.727**	**6.096**	**88.30**	**97.56**

**Table 8:** Interpretable performance comparison of significant and weak feature samples under different models in the CowBack dataset.

Models	Image1	Grad-CAM	Image2	Grad-CAM	Image Discription
EfficientnetV2	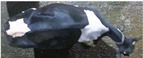	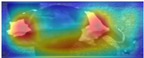	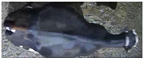	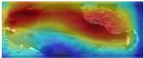	Original image
	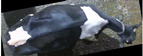	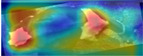	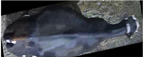	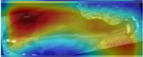	Random rotation
	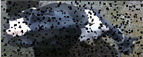	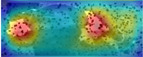	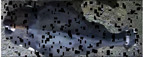	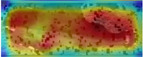	Random occlusion
	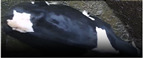	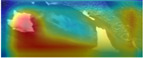	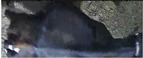	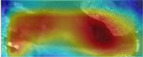	Different perspectives
EfficientnetV2 + ECA	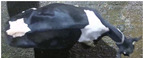	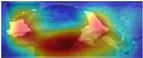	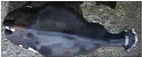	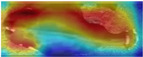	Original image
	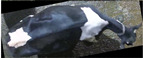	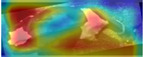	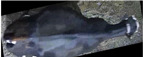	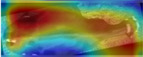	Random rotation
	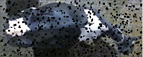	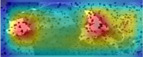	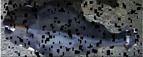	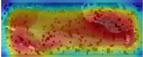	Random occlusion
	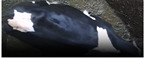	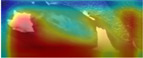	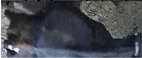	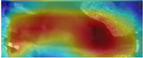	Different perspectives
CowBackNet (ours)	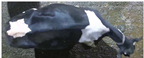	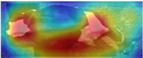	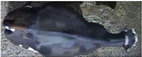	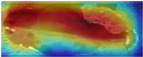	Original image
	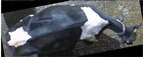	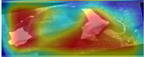	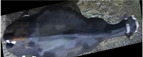	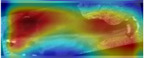	Random rotation
	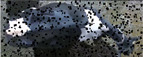	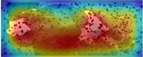	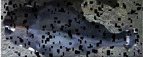	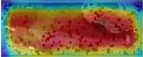	Random occlusion
	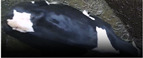	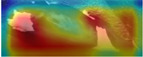	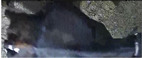	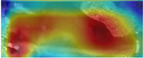	Different perspectives

**Table 9:** Interpretable performance comparison of significant and weak feature samples under different models in the Cows2021 dataset.

Models	Image1	Grad-CAM	Image2	Grad-CAM	Image Discription
EfficientnetV2	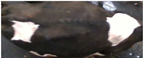	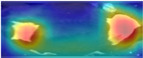	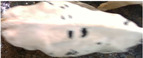	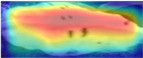	Original image
	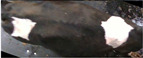	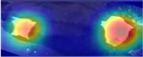	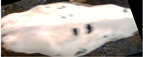	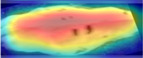	Random rotation
EfficientNetV2 + ECA	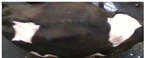	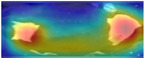	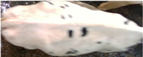	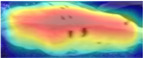	Original image
	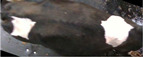	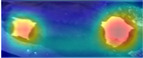	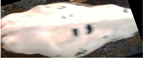	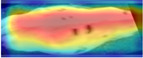	Random rotation
CowBackNet (ours)	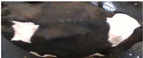	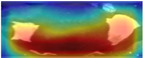	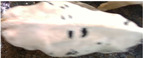	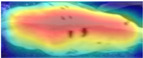	Original image
	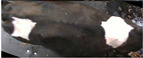	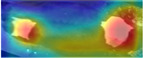	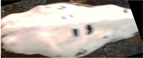	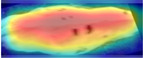	Random rotation

**Table 10:** Examples of misrecognized samples and reasons for misrecognition in CowBack test set.

Image	pred_label	gt_label	Cause	Image	pred_label	gt_label	Cause
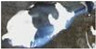	cow014	cow005	Interference in background	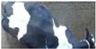	cow014	cow011	Different shooting angles
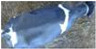	cow013	cow007	Image blurring	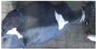	cow007	cow015	Interference in background
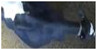	cow022	cow006	Different shooting angles	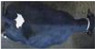	cow006	cow022	Image blurring

**Table 11:** Examples of misrecognized samples and reasons for misrecognition in Cows2021 test set.

Image	pred_label	gt_label	Cause	Image	pred_label	gt_label	Cause
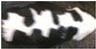	cow002	cow001	Image blurring	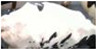	cow147	cow054	Interference in background
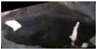	cow020	cow005	Loss of image information	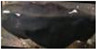	cow020	cow109	Low texture complexity
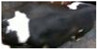	cow075	cow116	Interference in posture	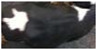	cow075	cow138	Image blurring
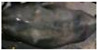	cow078	cow050	Low texture complexity	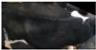	cow078	cow077	Loss of image information
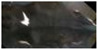	cow126	cow092	Loss of image information	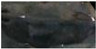	cow126	cow154	Low texture complexity

## Data Availability

The data presented in this study are available from the corresponding author upon reasonable request. The data are not publicly available due to privacy and confidentiality agreements that protect the commercial interests of the farms involved.

## Abbreviations

The following abbreviations are used in this manuscript:

ResNet	Residual Network
SSIM	Structure Similarity Index Measure
DSC	Deep Separable Convolution
NAS	Neural Network Architecture Search
SE	Squeeze-and-Excitation
CBAM	Convolutional Block Attention Module
ECA	Efficient Channel Attention
CA	Coordinate Attention
Grad-CAM	Gradient-weighted Class Activation Mapping
